# Quad-Level Cell Switching with Excellent Reliability in TiN/AlOx:Ti/TaOx/TiN Memory Device

**DOI:** 10.3390/ma15072402

**Published:** 2022-03-24

**Authors:** Hee Ju Shin, Hyun Kyu Seo, Su Yeon Lee, Minsoo Park, Seong-Geon Park, Min Kyu Yang

**Affiliations:** 1 Artificial Intelligence Convergence Research Lab, Sahmyook University, Seoul 01795, Korea; godqhr7801@gmail.com (H.J.S.); seohyunkyu0811@gmail.com (H.K.S.); chocotndus@gmail.com (S.Y.L.); xiurek@syu.ac.kr (M.P.); 2 Semiconductor Research & Development, Samsung Electronics, Hwaseong 18448, Korea; parksg1104@gmail.com

**Keywords:** ReRAM, resistive switching, ISPP, QLC

## Abstract

TiN/AlO_x_:Ti/TaO_x_/TiN memory devices using bilayer resistive switching memory demonstrated excellent durability and capability of QLC (quad-level cell) memory devices. The best nonvolatile memory characteristics with the lowest operation current and optimized 4 bit/cell states were obtained using the Incremental Step Pulse Programming (ISPP) algorithm in array. As a result, a superior QLC reliability (cycle endurance > 1 k at each level of the QLC, data retention > 2 h at 125 °C) for all the 4 bits/cell operations was achieved in sub-μm scaled RRAM (resistive random access memory) devices.

## 1. Introduction

The paradigm shift from a planar to vertical 3D structure of modern electronic devices is not a choice anymore. In the nonvolatile memory field, a 3D stackable crossbar-type ReRAM (resistive switching random access memory) device has been considered as the next-generation stand-alone memory device due to its simple structure, wide range of operational currents and fast write/erase speed. However, manufacturing high-density crossbar resistive memory cannot be realized simply by stacking multi floors or the scaling of device technology because the technology enablement unavoidably results in a rapid increase in the cost of device fabrication [1]. In this context, its main advantage over flash is that it is both more durable and requires far less energy per write. At present, long-term NAND flash scaling faces a number of problems, such as a flash built on smaller process nodes being less durable. An extension from MLC (multi-level cell) to TLC (triple level cell) has only been acceptable to a few limited applications due to the drastically reduced number of write cycles that a TLC flash can perform [2,3,4]. The QLC (quad levelcell) is still questionable. In theory, a resistance switching memory could solve a number of these problems. In this research, highly reliable ReRAM cells with QLC characteristics were fabricated using a bilayer structure for cross-point memory applications. Reproducible QLC behavior was successfully observed and elucidated by an oxygen ion migration model. Moreover, a new programming algorithm was developed for a more reliable and uniform QLC operation [5,6,7,8,9]. Recently, in-memory processing applications have attracted attention as cutting-edge chip technology that not only stores data in memory, but can also perform data operations. Accordingly, there is high interest in the resistive switching (RS) of various metal oxide materials for use in the implementation of in-memory computing utilizing next-generation, nonvolatile memory [10,11,12,13,14,15,16,17,18,19,20,21,22,23,24]. In this situation, as the amount of user data grows exponentially, low-energy operation is essential. To this end, new devices must meet high operating speeds, low operating voltages and high reliability. As a candidate to meet these requirements, RS random access memory (RRAM) shows excellent performance in terms of speed and reliability [25,26]. However, many RRAM studies conducted so far have been conducted in the high-current region (over 10 μA), making them unsuitable for future demanding electronic device applications [27,28,29]. The reason is that the reliability of the RRAM resistance state becomes difficult to achieve as the operating current decreases [30,31,32,33]. Approaches are being undertaken to overcome the reliability issue by adopting several types of electrodes or by changing the stacked structure [34,35], but there are still difficulties. In this study, we used Ti-doped alumina (Ti: Al_2_O_3_, TAO) and TaO_x_ as the RS and oxygen reservoir, respectively. Our device showed 4-bit characteristics and stable retention characteristics (125 °C, 2 h) at a low operating current (<1 μA). Ti doped into the RS layer played a central role in the generation of oxygen vacancies, resulting in excellent and gradual RS behavior. TaO_x_, used as an oxygen storage layer, also acts as an external load resistor to help achieve multibit operation and reliable retention characteristics.

## 2. Materials and Methods

The manufactured RS devices had a crossbar structure, and the cell size was 100 nm^2^. We patterned TiN (Pioneer Materials, Chongqing, China) as the bottom electrode (BE) by e-beam lithography and photolithography. TAO layer was deposited by the atomic layer deposition (ALD) technique while maintaining a temperature of 200 °C. Trimethyl aluminum (TMA, Merck, Darmstadt, Germany), titanium isopropoxide (TTIP, Merck, Darmstadt, Germany) and H_2_O were used as sources of Al, Ti and O, respectively. TiO_2_ and Al_2_O_3_ were alternately deposited in super cycle ALD to form a 5 nm thick TAO layer. A 20 nm thick TaO_x_ layer was deposited using a radio-frequency (RF) reactive sputtering method, of which the oxygen flow rate was 5 sccm. The base pressure for sputtering was 5 × 10^−7^ torr, and the working pressure was 1 × 10^−3^ torr. After TaO_x_ layer formation, TiN top electrodes (TE) were patterned using the same process as used for the BE, and the TE and BE were crossed perpendicularly to make the crossbar-type device. The thermal annealing test was conducted using furnace equipment to check retention performance by baking at 125 °C for 2 h. To check the amount of Ti in AlO_x,_ compositional analyses of the TiN/AlO_x_:Ti structure were performed based on the depth profile obtained via Auger electron spectroscopy (AES, PHI-700 ULVAC-PHI, Kanagawa, Japan). The electrical properties of our RS devices were tested using a semiconductor parameter analyzer (SPA, Keithley 4200 SCS, Beaverton, OR, USA) and arbitrary function generator (AFG, Agilent 81150A, Beaverton, OR, USA). The RF circuit-switching module accessed the two-terminal electric circuits between the SPA and the AFG, alternately. During all measurements, the TE was biased, while the BE was grounded. During multibit measurement, we used ISPP and the error check and correction (ECC) method (details concerning these methodologies available elsewhere).

## 3. Results

**Figure 1:**
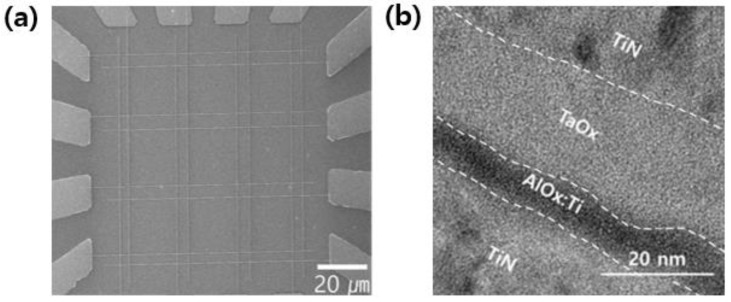
(**a**) SEM image from the top of TiN/AlO_x_:Ti/TaO_x_/TiN device. (**b**) TEM image of TiN/AlO_x_:Ti/TaO_x_/TiN.

**Figure 2:**
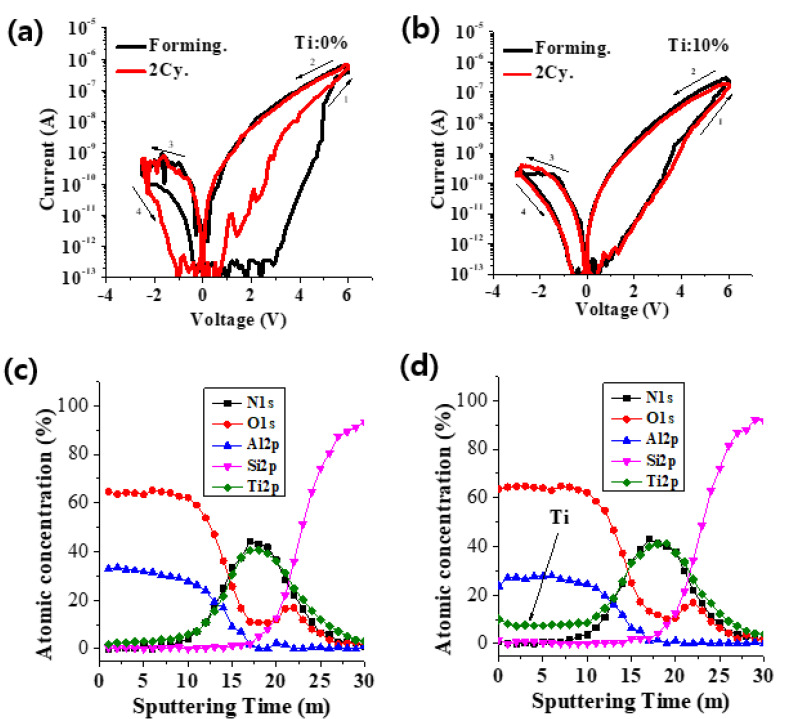
(**a**,**b**) show the DC I–V characteristics of the Ti 0% and 10% RRAM devices, respectively. (**c**,**d**) show the AES depth profile of devices.

Figure 1a,b shows the SEM (ZEISS, Jena, Germany) and TEM (JEOL, Akishima, Japan) image of TiN/AlO_x_:Ti/TaO_x_/TiN memory devices. The thickness of the AlO_x_ film was approximately 5 nm and the thickness of the TAO was confirmed to be 20 nm, respectively. Figure 2a,b shows the I–V characteristics according to the Ti 0% and 10% doping of Al_2_O_3_, which was the RS layer, respectively. To prevent a permanent dielectric breakdown, the compliance current (CC) was set to 1 uA during the ‘SET’ (resistance change from high-resistance state (HRS) to the low-resistance state (LRS) by external bias) measurement. The Ti 10%-doped Al_2_O_3_ device showed a very uniform distribution of the SET operating voltage at ~6 V and a RESET (resistance change from LRS to HRS) voltage distribution between −2.5 V and −3 V. Figure 2c,d shows the depth profile of AES to analyze the amount of Ti inside the AlO_x_ thin film of both devices. As a result of the analysis, it was confirmed that about 10% of Ti was doped inside AlO_x_, which would have improved the RS curve as well as other properties.

**Figure 3:**
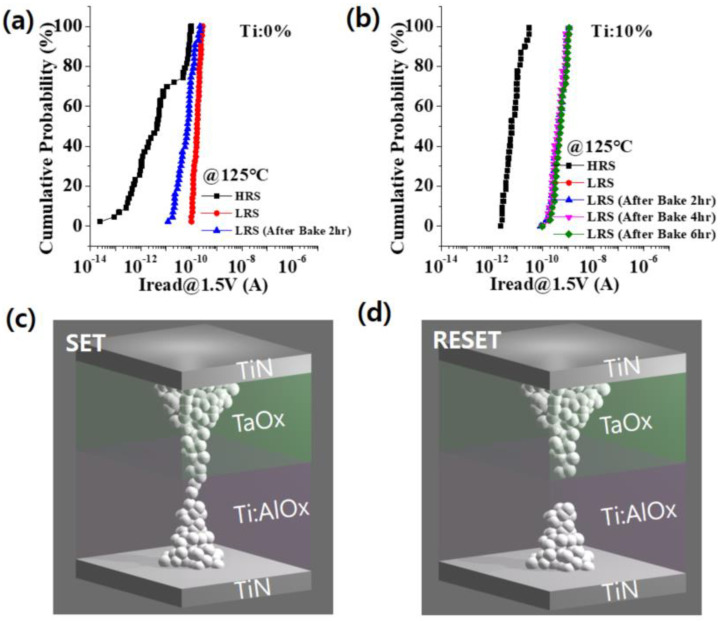
(**a**,**b**) show the retention characteristics of the Ti 0% and 10% RRAM devices, respectively. The schematic figures for a sequential RS cycle. (**c**,**d**) show the LRS the HRS of the RRAM device, respectively.

Figure 3a,b compares the retention of RRAM devices doped with Ti 0% and Ti 10%, respectively. They were baked at 125 °C for 2 h and, as a result of the before and after comparison, it was observed that the retention of the LRS of the device doped with Ti 0% was deteriorated. However, in the device doped with Ti 10%, it was observed that the LRS was stably maintained after 6 h. As a result, the Ti in AlO_x_ strengthened the retention of the device. Figure 3c,d schematically shows the mechanism of the RRAM device. The RS cycle started in the initial state. When a positive voltage was applied to the upper electrode (TE), oxygen ions moved from the resistive switching layer (Ti:AlO_x_) to the oxygen ion reservoir layer (TaO_x_) in Figure 3c. As a result, conduction paths were formed, resulting in an LRS state. The device then transitioned to HRS (Figure 3d) via a RESET operation. Then, the oxygen ions came out of the reservoir layer and the conduction paths were broken. However, the initial resistance of the primordial device was not fully recovered in HRS, indicating that some of the metal phase connections may have been lost and the electrically conductive paths may have been completely blocked by recombination with oxygen during RESET.

**Figure 4:**
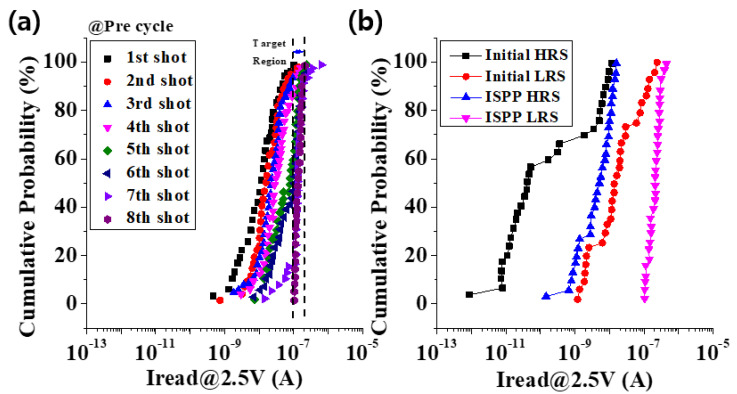
(**a**) Shows that the initial resistance states moved to the desired target range by the pre-cycle. (**b**) Comparison before and after applying ISPP algorithm.

As shown in Figure 4, the cells subjected to the ISPP pre-cyclic pulse had a narrower distribution of resistance states. Compared to the initial state cells that did not use the ISPP pre-cycling method, cells to which ISPP pre-cycling was applied were highly likely to show an improved performance of the 4-bit/cell operation. A uniformly formed microresistive switching region in a cell could be considered to benefit from a uniform and stable performance. 

**Figure 5:**
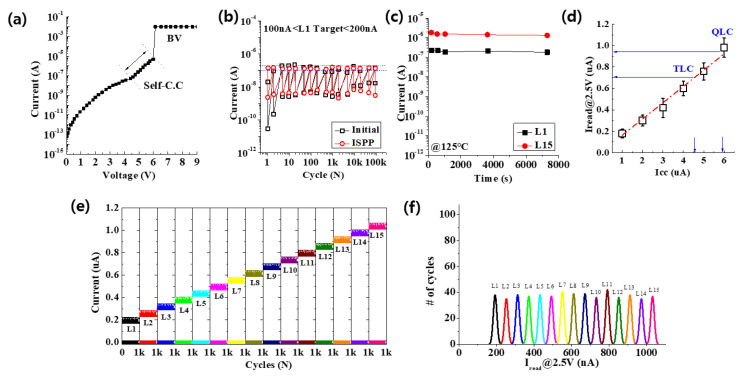
(**a**) Shows the self-compliance effect and the BV. (**b**) Endurance of the initial cell and the cell to which ISPP algorithm was applied. (**c**) Retention of Level 1 and Level 15 of the LRS state. (**d**) Current range per bit. (**e**) Shows the endurance of 1 k cycle for each level. (**f**) Distributions of the 4 bits/cell operation using the ISPP in array.

For the current limiting the application of an electrical pulse, a cell-specific ‘self-compliance’ effect was introduced, as shown in Figure 5a. After the SET operation of the cell, the magnetic compatibility region could function as an external resistor to control the sudden increase in the current. As a current-limiting device, the self-compliance effect helped achieve stable and reliable multibit performance without electrical errors during the ISPP operation. It was confirmed that applying the electric pulse pre-cycle method to a clean cell was effective in improving the device’s operation yield, minimizing the number of pulses (NOP), and improving the resistance-state distribution. Figure 5b is the result of measuring the endurance of the cell and the initial cell to which the ISPP algorithm was applied to for 100 k cycles. As a result of applying the ISPP algorithm targeting the Level 1 current range, it was confirmed that it was perfectly within the desired range current. This method was then applied to obtain a 4-bit result. Figure 5c confirms the retention of Level 1 and Level 15 of the LRS state. As a result, stable retention was confirmed at 125 °C for 7200 s. In RRAM operating with a low current, finding a stable current range of Level 1 was a very important part of the multi-bit test. Figure 5d shows that the experimental pulse switching and simulation results agreed, which shows how many multi-bits could be divided. Figure 5e is the result of dividing from Level 1 to Level 15, while showing an endurance of 1 k cycles for each level. At this time, the HRS state was safely maintained. Figure 5f shows the distributions in 4 bits/cell operation using the ISPP verifying in array. The tested data were characterized at 1 k SET/RESET cycles, which was within the nominal lifetime of the experimental RRAM memory. In qualifying the state overlap probability (SOP) between the resistance states, we assumed that the obtained resistance values obeyed a Gaussian distribution, and evaluated the SOP values using the equation $\sigma =\frac{m_{2}-m_{1}}{\mu_{2}-\mu_{1}}$, where *m*_2_, *m*_1_, *μ*_2_, and *μ*_1_ represent the average and standard deviations between neighboring resistance states, respectively. This equation implies that the larger the value of *σ*, the smaller the SOP. The distribution of each state was distinguishable from one another (lower than 2.45σ (0.71%) overlap in the worst case of the 4 bits/cell). The data indicated an excellent reliability performance in the 4 bits/cell RRAM devices. 

## 4. Conclusions

By using the ISPP algorithm, we successfully achieved a 4 bits/cell approach in the sub-μm scaled RRAM devices in array. The sub-μm scaled RRAM devices exhibited a superior QLC reliability (cycle endurance > 1 k @each level, data retention > 2 h at 125 °C). Higher Ti concentrations in the TAO layer generated more leakage current characteristics and were not suitable for a stable RS operation. The SET and RESET operating voltages must still be high to facilitate a low-power RS operation depending on energy dependency. However, the QLC properties achieved in nonvolatile memory may provide guidance for the resistive switching of various metal oxide materials to be used in the implementation of in-memory computing utilizing next-generation, non-volatile memory in the future.

## Data Availability

Not applicable.
